# Categorization of the effects of *E. coli* LF82 and mutants lacking the *chuT* and *shuU* genes on survival, the transcriptome, and metabolome in germ‐free honeybee

**DOI:** 10.1002/2211-5463.13776

**Published:** 2024-02-25

**Authors:** Dongping Feng, Hujun Zhang, Zhengpeng Li, Yiyuan Li, Jingshuang Yan, Yan Zhang, Yunsheng Yang

**Affiliations:** ^1^ Microbiota Division, Department of Gastroenterology and Hepatology The First Medical Center, Chinese PLA General Hospital Beijing China; ^2^ Medical School of Chinese PLA Beijing China; ^3^ Institute of Genetics and Developmental Biology Chinese Academy of Sciences Beijing China

**Keywords:** AIEC LF82, *chuT*, CRISPR/Cas9 system, inflammatory bowel disease, iron uptake, *shuU*

## Abstract

The precise etiology of inflammatory bowel diseases (IBDs) remains elusive. The *Escherichia coli* strain LF82 (LF82) is known to be associated with IBD, and we hypothesized that this association may be related to the *chuT* and *shuU* genes. Here we constructed a germ‐free (GF) honeybee model to investigate the effects of LF82 *chuT* and *shuU* genes on the honeybee intestine and their mechanisms. The *chuT* and *shuU* gene deletion strains LF82∆chuT and LF82∆shuU were generated by CRISPR‐Cas9. These strains, together with nonpathogenic *E. coli* MG1655 (MG1655) and wildtype LF82, were allowed to colonize the guts of GF honeybees to establish single bacterial colonization models. Intestinal permeability was assessed following the administration of a sterile Brilliant Blue (FCF) solution. Comprehensive transcriptomic and metabolomic analyses of intestinal samples indicated that MG1655 had few disadvantageous effects on honeybees. Conversely, colonization with LF82 and its gene‐deletion mutants provoked pronounced activation of genes associated with innate immune pathways, stimulated defensive responses, and induced expression of genes associated with inflammation, oxidative stress, and glycosaminoglycan degradation. Crucially, the LF82∆chuT and LF82∆shuU strains perturbed host heme and iron regulation, as well as tryptophan metabolism. These findings suggest that the deletion of *chuT* and *shuU* genes in *E. coli* LF82 may alleviate intestinal inflammation by partially modulating tryptophan catabolism. Our study proposes that targeting iron uptake mechanisms could be a potential strategy to mitigate the virulence of IBD‐associated bacteria.

## Graphical abstract

The iron derived from heme is essential for the survival and virulence of bacteria. The deletion of *chuT* or *shuU* in *Escherichia coli* LF82 perturbed host heme and iron regulation, as well as tryptophan metabolism. Our study proposes that targeting iron uptake mechanisms could be a potential strategy to mitigate the virulence of inflammatory bowel disease‐associated bacteria.

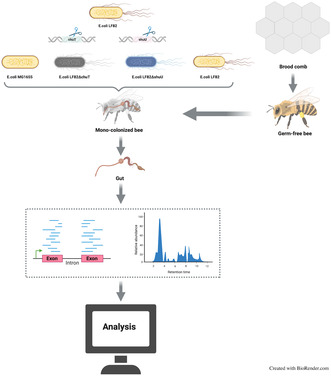

**Abbreviations**
ABC: ATP‐binding cassette transporter
AIEC: adherent‐invasive *Escherichia coli*
bp: base pair
CD: Crohn's disease
DEGs: differentially expressed genes
GAGs: glycosaminoglycans
GF: germ‐free
GO: Gene Ontology
gRNA: guide RNA
GSH: glutathione
IBD: inflammatory bowel disease
KEGG: Kyoto Encyclopedia of Genes and Genomes
LB: Luria broth
LC–MS: liquid chromatography‐mass spectrometry
LF82: *Escherichia coli* strain LF82
OD: optical density
PCA: principal components analysis
PLS‐DA: partial least squares discrimination analysis
QC: quality control
ROS: reactive oxygen species
rpm: revolution per minute
VIP: variable importance in the projection

Inflammatory bowel disease (IBD) is a chronic, relapsing inflammatory condition of the intestine, primarily comprising two subtypes: Crohn's disease (CD) and ulcerative colitis [1]. Epidemiological data indicate that IBD, a disease with a rising global incidence, exhibits regional prevalence variations [2, 3]. By 2025, the population of IBD patients will exceed 1.5 million in China [2, 4, 5].

Although significant progress has been made in elucidating the pathogenesis of IBD and expanding therapeutic options, the precise etiology of IBD remains elusive, involving a complex interaction of genetic, immunological, environmental factors, intestinal barrier integrity, gut microbiota balance, and potential pathogens [6]. Especially, it is harmful that virulence factors of pathogenic bacteria, such as iron acquisition systems, invasive factors, and capsules to hosts [7]. Bacterial iron metabolism, particularly heme uptake, is crucial for bacterial survival and virulence [8]. Subsequently, after binding to bacterial outer membrane receptors and passing through the outer membrane of cells to periplasmic binding protein, heme enters the cytoplasm through the ABC transporter into the inner membrane for bacterial iron metabolism [8].

The adherent‐invasive *Escherichia coli* (AIEC), the representative strains *E. coli* LF82, is associated with IBD pathogenesis [9, 10, 11, 12]. In pathogenic *E. coli*, the *chu* gene cluster regulates the heme uptake system, encoding proteins for heme transport and processing. The *shuU* is homologous to the *chuT* gene and encodes ABC transporter permease, which is implicated in heme transport in *Shigella* and pathogenic *E. coli*, respectively [13].

This study employed CRISPR‐Cas9‐mediated gene editing to knock out the *chuT* and *shuU* genes in *E. coli* LF82, respectively. The honeybee (*Apis mellifera*), a model organism [14, 15] with a well‐characterized genetic background, complete omics databases, and a simple gut microflora, serves as an ideal germ‐free (GF) model for investigating gut microbiology. Additionally, honeybees' short developmental and reproductive cycles, coupled with low maintenance costs, further underscore its utility in research. Our research focuses on elucidating the mechanisms of the *chuT* and *shuU* genes in the LF82 strain, thus enriching the current understanding of IBD pathogenesis.

## Materials and methods

### Construction of recombinant plasmids and gene editing

Following the methodology of Li *et al*. [16], we obtained the pEcgRNA plasmid (#166581) and the pEcCas plasmid (#73227) from Addgene (https://www.addgene.org). We designed guide RNAs (gRNAs) targeting the genes *chuT* and *shuU*, referred to as LF82 chuT‐sgRNA and LF82 shuU‐sgRNA, respectively, using the CHOPCHOP web tool (http://chopchop.cbu.uib.no). We synthesized the corresponding oligonucleotides and their reverse complementary fragment. The pEcgRNA plasmid was linearized with *Bsa*I endonuclease, and the single‐stranded sgRNAs were annealed to generate double‐stranded sgRNAs. These were subsequently ligated into the linearized pEcgRNA plasmid to construct the recombinant plasmids pEcg‐LF82‐∆chuT and pEcg‐LF82‐∆shuU, respectively.

To generate homologous arms for the *chuT* and *shuU* genes, we amplified chuT‐up, chuT‐dw, shuU‐up, and shuU‐dw fragments by PCR using TransStart FastPfu Fly DNA Polymerase. The recombinant plasmids pEcg‐LF82‐∆chuT and pEcg‐LF82‐∆shuU were linearized with *Hind*III endonuclease. The triple fragment ligation was then performed, employing NovoRec recombinase to join the linearized pEcgRNA plasmids with chuT‐up and chuT‐dw or shuU‐up and shuU‐dw. The resulting recombinant plasmids were named pEcgT‐LF82‐∆chuT and pEcgT‐LF82‐∆shuU. These new plasmids were chemically transformed into engineering bacteria DH5α before spreading plate and monoclonal sequencing. The pEcCas plasmid was also transformed into the LF82 strain, yielding the LF82/pEcCas construct. The pEcgT‐LF82‐∆chuT, pEcgT‐LF82‐∆shuU, and LF82/pEcCas plasmids were utilized in subsequent experiments following verification of the correct sequences.

#### Gene editing

LF82/pEcCas plasmids were co‐transformed with either pEcgT‐LF82‐∆chuT or pEcgT‐LF82‐∆shuU into the LF82 strain. Following successful gene knockout, the plasmid ablation operation was performed.

The primers used in these experiments are listed in the Table S1.

### Generation of germ‐free and mono‐colonized honeybees

Honeybees (*A. mellifera*) used in this study were from colonies maintained in the experimental apiary of Jinhaihu‐huanle in Pinggu District, Beijing. Late‐stage pupae emerged bees used in our experiments were obtained manually from brood frames and kept in an incubator at 35 °C, with humidity of 50%. There is no current requirement regarding insect care and use in research. Experimental honeybees were fed daily with pollen and sucrose solutions during the experimental period. Newly emerged bees (day 0 of bee age) were given 50% sterile sucrose (wt/vol). Each experimental group fed the bees after adding the bacterial solution (OD600 = 1) to sterile sucrose separately, and strain colonization was determined by bacterial colonization test.

### Samples tissue collection

The experimental bees were humanely euthanized with carbon dioxide anesthesia before being delicately dissected. Holding the bees securely in the left hand, we used sterile metal forceps in the right hand to gently grasp and extract the intestines, simultaneously removing the tail poison gland. Then the harvested intestines were promptly placed into 1.5 mL nuclease‐free centrifuge tubes and then immediately submerged in a liquid nitrogen tank. Finally, following the collection of all intestinal samples, they were collectively transferred from the liquid nitrogen to a −80 °C freezer for preservation.

### DNA and RNA extraction

#### DNA extraction

The genomes extraction of the experimental strains was performed by using the TIANGEN (TIANGEN Biotech Co., Ltd., Beijing, China) Centrifuge Column Type Bacterial Genomic DNA Extraction Kit (DP302), following the instructions of the kit, and the DNA concentration was measured after completion.

#### RNA extraction

Total RNA was isolated from experimental honeybees' samples of each group using TRIzol™ Reagent Invitrogen (Thermo Fisher Scientific Inc, Waltham, MA, USA). Each dissected gut was homogenized with a plastic pestle, and total RNA was extracted from individual samples according to the manufacturer's protocols (74104; QIAGEN, Venlo, The Netherlands).

### Analysis of transcriptomics data

#### RNA sequencing

Total RNA from all samples was sent to Novogene Bioinformatics Technology Co. Ltd., Beijing, China, for transcriptome sequencing. RNA integrity was verified using the RNA Nano 6000 Assay Kit on the Bioanalyzer 2100 system (Agilent Technologies, Santa Clara, CA, USA). Sequencing libraries were prepared using a universal method and evaluated with the Qubit 2.0 Fluorometer (Life Technologies, Waltham, MA, USA) and the Agilent 2100 bioanalyzer. Sequencing was conducted on the Illumina Novaseq 6000 platform (Illumina, San Diego, CA, USA), producing 150 bp paired‐end reads. The honeybee reference genome (Amel_HAv3.1) and annotations were obtained from NCBI (https://www.ncbi.nlm.nih.gov/genome/). hisat2 (v2.0.5) was used for genome indexing and read alignment. stringtie (v1.3.3b) assembled the reads, and featurecounts (v1.5.0‐p3) quantified gene read counts.

#### RNA‐seq analysis

Initial data processing (quality control, alignment, quantification) was executed on a Linux server using the Amel_Hav3.1 honeybee genome. Subsequent analyses were conducted with r (R‐4.2.0, Vienna, Austria) and cytoscape (3.9.1). Differential expression analysis was performed using deseq2 (v1.38.3), while GO and KEGG enrichment analyses utilized clusterprofiler (v4.7.1.002), based on the hypergeometric distribution. Protein interaction networks for differentially expressed genes (DEGs) were analyzed using the STRING database.

### Analysis of metabolomics data

Seven biological replicates from each experimental group were subjected to untargeted metabolomics analysis. The liquid chromatography‐mass spectrometry (LC–MS) analyses was carried out by Novogene Co., Ltd. (Beijing, China). The work was conducted using a Vanquish UHPLC system (Thermo Fisher Scientific Inc) coupled with an Orbitrap Q Exactive™ HF mass spectrometer (Thermo Fisher Scientific Inc), operating in positive/negative polarity mode. For data processing, the raw UHPLC–MS/MS outputs were analyzed using compound discoverer 3.1 software (Thermo Fisher Scientific Inc), which facilitated the accurate identification and relative quantification of metabolites. Subsequent metabolomic data analysis was performed using the metaboanalyst (version 5.0, Montreal, Canada) interface and r (R‐4.2.0) software.

These metabolites were annotated using the KEGG database, HMDB database (https://hmdb.ca/metabolites) and LIPIDMaps database (http://www.lipidmaps.org/). Principal components analysis (PCA) and Partial least squares discriminant analysis (PLS‐DA) were performed using r (R‐4.2.0) software. We applied univariate analysis (*t*‐test) to calculate the statistical significance (*P*‐value). The metabolites with VIP > 1 and *P*‐value < 0.05 and fold change ≥ 2 or FC ≤ 0.5 were differential metabolites.

### Correlation analysis of transcriptomic and metabolomic data

In this research, we conducted a bi‐omics correlation analysis, employing both Pearson and Spearman methods, to assess the relationships between genes and metabolites. Utilizing the r package wgcna (v1.72‐1), we identified significant gene‐metabolite pairs with *R* > 0.91 and *P*‐value < 0.05. These pairs were then visualized in the nine‐quadrant plots for each experimental group. Additionally, we constructed a network integrating transcriptomic and metabolomic data. Correlation coefficients for this network were computed using the r package psych (v2.3.3), and the network visualization was generated with the r package igraph (v1.4.2). The analysis focused on KEGG‐annotated genes and their corresponding metabolites, which were jointly enriched in both the transcriptome and metabolome.

## Results

### *E. coli* LF82 *chuT* and *shuU* gene editing

**Fig. 1:**
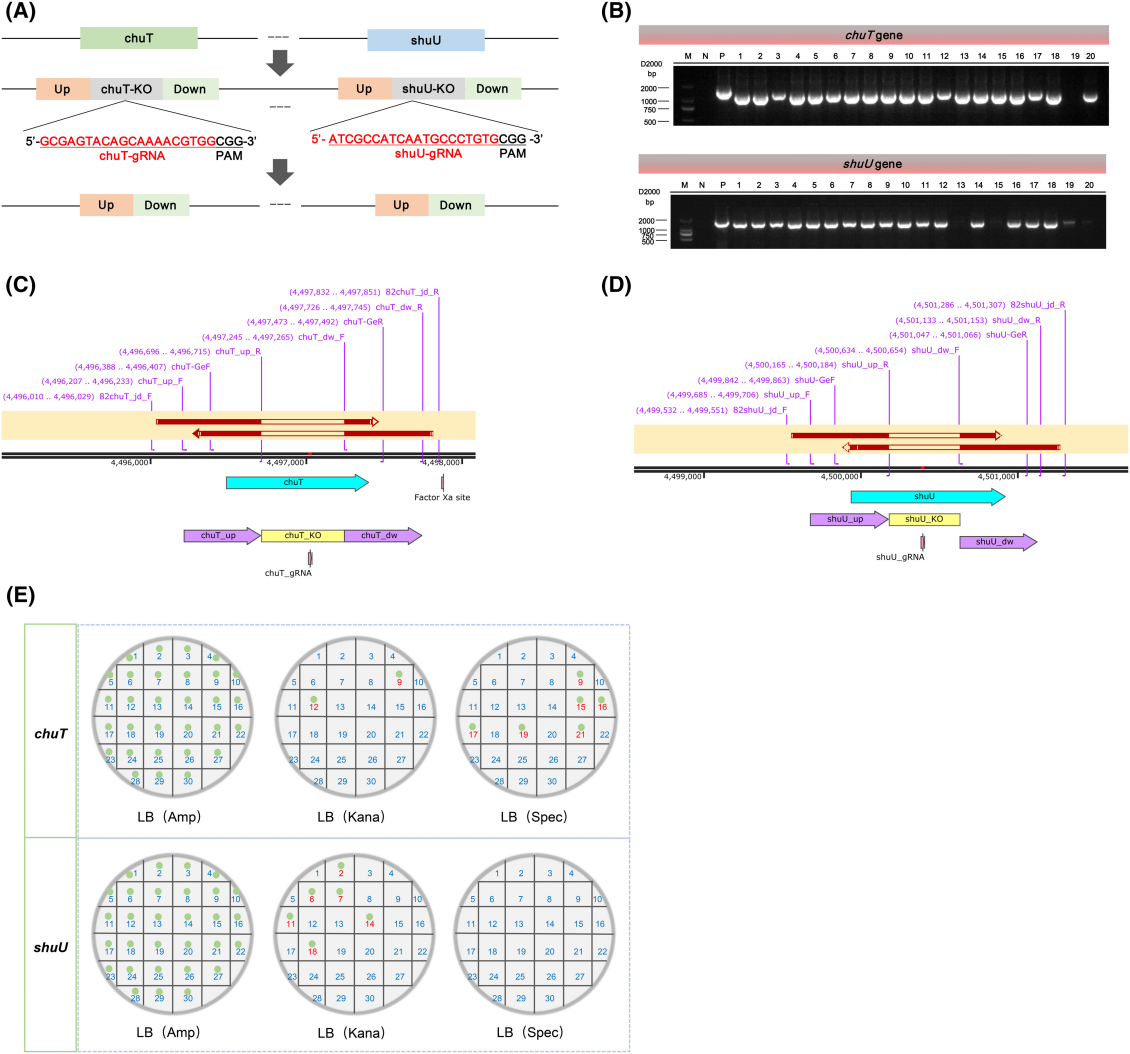
CRISPR‐Cas9‐mediated knockout of *Escherichia coli* LF82 *chuT* and *shuU* genes. (A) Diagram detailing CRISPR‐Cas9 gene editing: gRNAs guide Cas9 to *chuT* and *shuU* gene sites (red) for targeted cleavage next to PAM sequences (black). Homologous arms (up and down) facilitate knockout fragment (KO) recombination for *chuT* and *shuU*; up, up homologous arm; down, down homologous arm; chuT‐KO, deletion fragments of *chuT*; shuU‐KO, deletion fragments of *shuU*. (B) Validation of *chuT* and *shuU* gene knockouts using Agarose Gel Electrophoresis: ‘M’ indicates Molecular size marker (Marker II); ‘N’ is the negative control; ‘P’ is the positive control; Lanes 1, 2, 4–7, 8–11, 13–16, 18 show bands indicating successful *chuT* editing; Lanes 1–2, 3–11, 12, 14, 16, 17–18 confirm *shuU* editing. (C,D) Sequencing alignments of PCR products confirm specific gene editing in *chuT* and *shuU*. (E) A scheme of bacterial growth plates, whose original image is shown in Fig. S1. Plasmid curing verified by the one‐step method: IDs 1–8, 11, 13–14, 18, 20, 22–30 indicate pEcgT‐LF82‐∆chuT and pEcCas were removed successfully (above); IDs 1, 3–5, 8–10, 12–13,15–17, 19–30 indicate pEcgT‐LF82‐∆shuU and pEcCas were removed successfully (below). Green dots represent clones; antibiotic resistance markers for selection are Amp (ampicillin), Kan (kanamycin), and Spec (spectinomycin).

In this study we applied a CRISPR‐Cas9 system‐mediated gene editing approach to complete the knockout of the target genes *chuT* and *shuU* using the pEcCas/pEcgRNA system (Fig. 1A). The pEcgT‐LF82∆chuT plasmid, containing the gRNA and repair template, along with the pEcCas plasmid, were transformed into the *E. coli* LF82 to achieve *chuT* gene knockout. This editing resulted in a 530 bp deletion, confirmed by PCR and sequencing. The efficiency of *chuT* gene editing was 70% (14/20 clones) To remove the exogenous plasmid, we applied a one‐step plasmid curing method as described by Yang and colleagues, with a success rate of 77% (23/30 clones) (Fig. 1B,C,E) [16]. Similarly, we inactivated the *shuU* gene, generating 450 bp deletion with an editing efficiency of 80% (16/20 clones) and a plasmid curing efficiency of 80% (24/30 clones) (Fig. 1B,D,E). In brief, the pEcCas/pEcgRNA system effectively generated *E. coli* LF82 strains with deletions in the *chuT* or *shuU* genes, yielding the LF82∆chuT and LF82∆shuU strains, as illustrated in Fig. 1.

### Survival duration and intestinal permeability

**Fig. 2:**
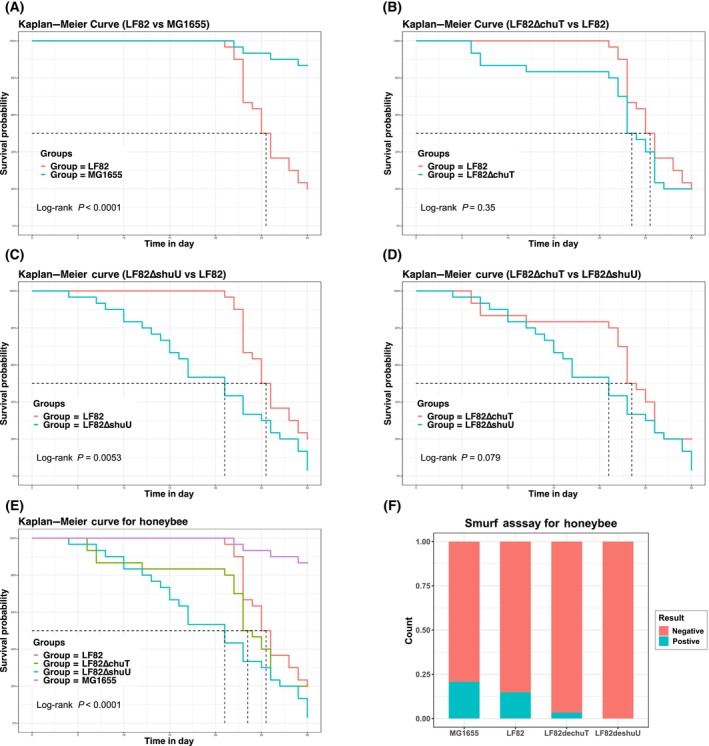
Comparative analysis of survival and intestinal integrity in honeybees following colonization by various *Escherichia coli* strains. (A–D) Display Kaplan–Meier survival curves comparing the longevity of *Apis mellifera* when colonized with different *E. coli* strains: wildtype LF82 versus nonpathogenic MG1655, LF82∆chuT versus LF82, LF82∆shuU versus LF82, and LF82∆chuT versus LF82∆shuU, respectively, illustrating differential survival outcomes linked to bacterial gene knockout. (E) Compiles the survival curves of all honeybee groups, providing an overarching view of survival rates across the experimental conditions. (F) Presents results from the Smurf assay, assessing intestinal mucosal barrier integrity in different bacterial colonization models to indicate the impact of each bacterial strain on the intestinal barrier.

To discern the effects of *E. coli* strains on hosts' survival and gut integrity, guts of sterile honeybees were colonized with strains MG1655, LF82, LF82∆chuT, and LF82∆shuU. Survival and intestinal permeability were assessed postcolonization. We employed the Kaplan–Meier method to analyze the honeybees' survival data for the MG1655 group, the LF82 group, and its mutant counterpart, and assessed the survival curves of all four groups using the log‐rank test. Our analysis revealed that bees in the MG1655 group exhibited a longer survival duration compared to the other groups, with the survival curves demonstrating statistically significant differences across the groups (*P* < 0.0001), as depicted in Fig. 2E. Subsequent pairwise comparisons revealed that bees in the LF82 group had a significantly reduced survival time compared to those in the MG1655 group (*P* < 0.0001, Fig. 2A), yet a longer survival time than bees in the LF82ΔshuU group (*P* = 0.0053, Fig. 2C). However, when comparing the survival curves of bees in the LF82ΔchuT group with those in the LF82 group and the LF82ΔshuU group, no statistical differences were observed (*P* > 0.05, Fig. 2B,D). The Smurf assay revealed variations in gut permeability (*P* = 0.041), yet no significant differences were detected postadjustment (*P* > 0.05) (Fig. 2F). These results contribute to our understanding of *E. coli*'s pathogenicity and its interaction with the bee gut microbiome.

### Effects of LF82 and its mutant strains on the transcriptome of the honeybee gut

**Fig. 3:**
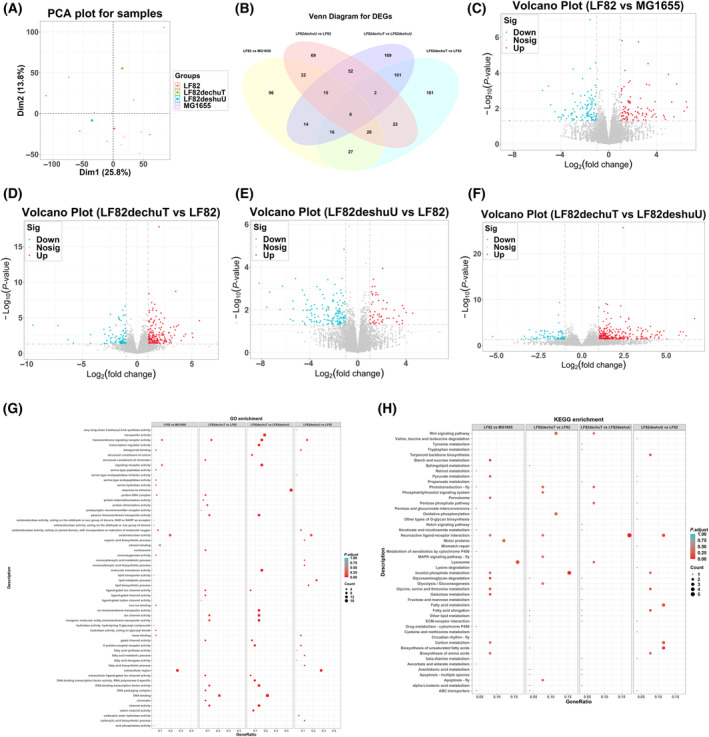
Transcriptomic analysis of *Apis mellifera* intestine response to *Escherichia coli* LF82 strains. (A) Principal components analysis (PCA) plot displays the transcriptional profile dispersion among samples. (B) A Venn diagram highlighting the overlap of DEGs among different bacterial colonization models. (C–F) Volcano plots delineate significant DEGs in honeybee guts for LF82 versus MG1655, LF82∆chuT versus LF82, LF82∆shuU versus LF82, and LF82∆chuT versus LF82∆shuU comparisons, respectively, highlighting genes with significant expression changes. (G) Gene ontology (GO) enrichment analysis comprehensively illustrates the varied biological processes (BP), molecular functions (MF), and cellular components (CC) most significantly impacted by each *E. coli* strain. The analysis showed that LF82 genes were primarily involved in carbohydrate metabolism, redox processes, oxidoreductase activity, and heme binding, similar to LF82∆chuT's upregulated genes compared to MG1655. LF82∆shuU's upregulated genes were enriched in carbohydrate metabolism, redox processes, oxidoreductase, and iron ion binding. (H) Kyoto Encyclopedia of Genes and Genomes (KEGG) pathway analysis identifies pathways and gene networks differentially engaged by the honeybees in response to each bacterial treatment; compared to MG1655, LF82, and its mutants (LF82∆chuT, LF82∆shuU) showed pathway enrichment in lysosome, glycosaminoglycan degradation, and Toll/Imd signaling. Besides, LF82∆chuT and LF82∆shuU were enriched in tryptophan metabolism and fatty acid degradation. LF82∆chuT specifically showed MAPK signaling and apoptosis pathway enrichment, while LF82∆shuU was enriched in the biosynthesis of unsaturated fatty acids and starch/sucrose metabolism.

In this study, RNA‐seq was applied to detect gene expression changes in the intestine of honeybees from different experimental groups (MG1655, LF82, LF82∆chuT, LF82∆shuU) with three biological replicates. After data quality control and reference genome alignment, quantitative gene expression count results were obtained for each sample (Fig. 3A). Utilizing the r package deseq2 (v1.38.3), we identified DEGs using thresholds of *P‐*value < 0.05 and log_2_ (Fold Change) > 1 or log_2_ (Fold Change) < −1. The range of DEGs between groups varied from 200 to 400 (Fig. 3B), and we drew corresponding volcano plots (Fig. 3C–F).

Functional enrichment analyses were conducted using the r package clusterprofiler (v4.7.1.002) for both Gene Ontology (GO) and KEGG pathways on the DEG sets. The top 20 most significant KEGG pathways are highlighted in bubble diagrams (Fig. 3G,H). GO enrichment analysis revealed that in the LF82 group, genes were predominantly associated with carbohydrate metabolic processes, oxidation–reduction processes, oxidoreductase activity, and heme binding, similar to the upregulated genes in the LF82∆chuT group when compared to MG1655. The LF82∆shuU group's upregulated genes were notably enriched in terms related to carbohydrate metabolism, oxidation–reduction processes, oxidoreductase activity, heme binding, and iron ion binding, etc. KEGG pathway analysis indicated that, relative to the MG1655 group, genes in the LF82, LF82∆chuT, and LF82∆shuU groups were enriched in pathways such as lysosome, glycosaminoglycan degradation, Toll and Imd signaling pathways, and pentose and glucuronate interconversions. Remarkably, genes in the LF82∆chuT and LF82∆shuU groups showed enrichment in tryptophan metabolism and fatty acid degradation pathways. When compared to the LF82 group, the LF82∆chuT group exhibited enrichment in the MAPK signaling and apoptosis pathways, while the LF82∆shuU group showed enrichment in the biosynthesis of unsaturated fatty acids and starch and sucrose metabolism pathways (Fig. 3G,H).

### Effects of LF82 and its mutant strains on the metabolome of the honeybee gut

**Fig. 4:**
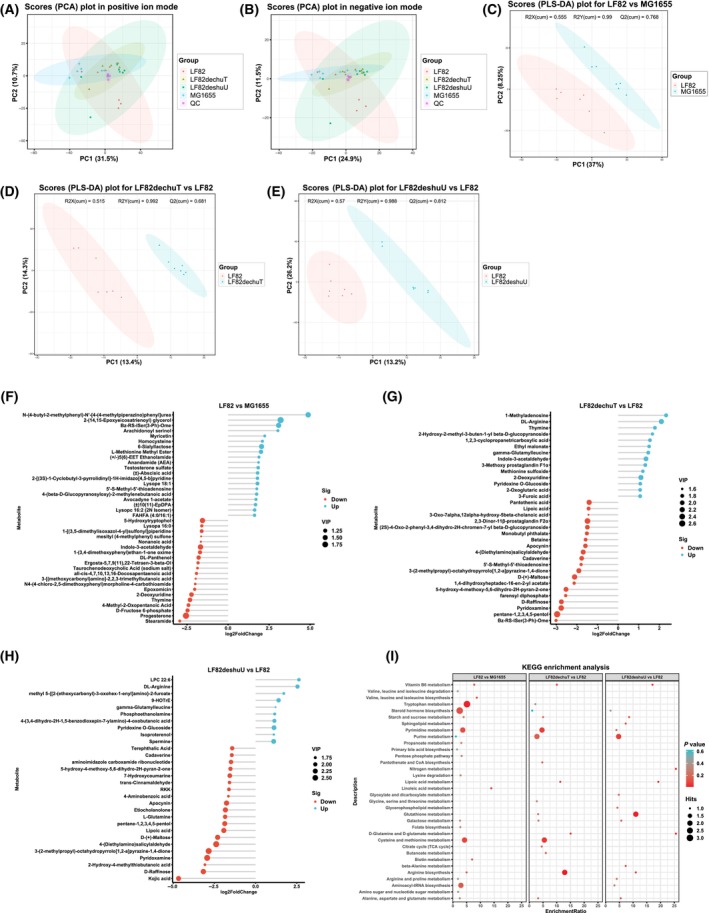
The gut metabolome analysis of *Apis mellifera*. (A,B) PCA plots delineate metabolic profiles in positive and negative ion modes, reflecting the overall metabolomic differences among the samples. (C–E) PLS‐DA plots contrasting LF82 with MG1655, LF82∆chuT with LF82, and LF82∆shuU with LF82, illustrating distinct metabolomic separations induced by gene deletions. (F–H) Lollipop plots highlight key differential metabolites between LF82 and MG1655, LF82∆chuT and LF82, and LF82∆shuU and LF82, thresholds of VIP > 1.0, FC > 2 or FC < 0.5, and *P*‐value < 0.05. (I) KEGG pathways analysis of significant differential metabolites in groups. KEGG enrichment analysis showed differential metabolite pathways: LF82 group in galactose, pyrimidine, tryptophan, and propionate metabolism compared to MG1655. LF82∆chuT displayed enrichment in glutathione and starch/sucrose metabolism versus LF82, while LF82∆shuU showed glutathione, carbon, ascorbic acid, and aldehyde metabolism.

In this investigation we utilized UHPLC–MS/MS for untargeted metabolomic profiling of the host intestine. Our comprehensive metabolome analysis identified a total of 1341 metabolites, including 968 in positive ion mode and 373 in negative ion mode. We conducted PCA on the relative quantitation of all experimental samples, including Quality Control (QC), in both ionization modes (Fig. 4A,B). Subsequent multivariate statistical analysis PLS‐DA demonstrated significant group separation. In comparisons between LF82 and MG1655, LF82∆chuT and LF82, and LF82∆shuU and LF82, the first principal component accounted for 37%, 13.4%, and 13.2% of the variance, respectively, with corresponding Q2 values of 0.768, 0.681, and 0.812, indicating robust model reliability (Fig. 4C–E).

For differential metabolite identification, we set thresholds of VIP > 1.0, FC > 2 or FC < 0.5, and *P*‐value < 0.05, and visualized these metabolites using lollipop (matchstick) plots (Fig. 4F–H). KEGG pathway enrichment analysis of differential metabolites revealed that, compared to the MG1655 group, the LF82 group's metabolites were primarily enriched in galactose metabolism, pyrimidine metabolism, tryptophan metabolism, and propionate metabolism pathways. Compared with the LF82 group, LF82∆chuT metabolites were predominantly enriched in glutathione metabolism and starch and sucrose metabolism, while LF82∆shuU metabolites were enriched in glutathione metabolism, carbon metabolism, as well as ascorbic acid and aldehyde metabolism pathways. When compared to the LF82∆shuU group, LF82∆chuT metabolites were mainly enriched in sphingolipid metabolism, purine metabolism, and other pathways (Fig. 4I). These findings provide valuable insights into the metabolic alterations associated with different *E. coli* strains in the honeybee intestine, with potential implications for understanding host–microbe interactions and intestinal health.

### Conjoint analysis of transcriptome and metabolome

**Fig. 5:**
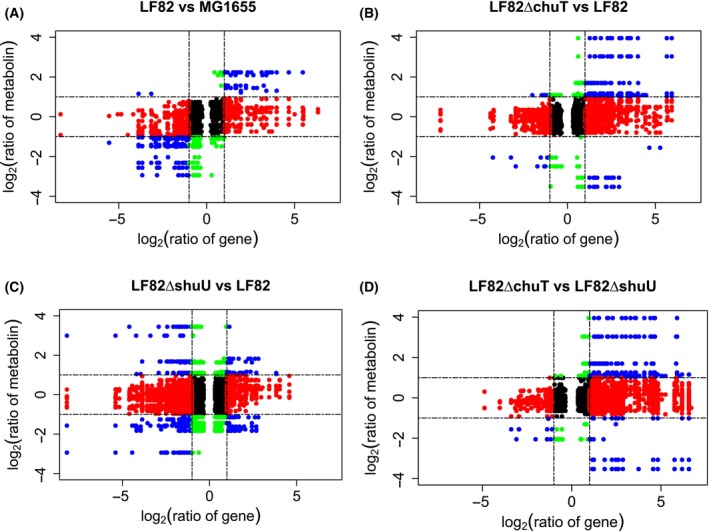
Results of the nine quadrant plots. (A–D) The nine quadrant plots of LF82 versus MG1655, LF82∆chuT versus LF82, LF82∆shuU versus LF82, and LF82∆chuT versus LF82∆shuU, respectively. The results indicate that *chuT* and *shuU* gene deletions in LF82 significantly affect its pathogenicity, with *chuT* causing notable variations and *shuU* being important for virulence. *shuU* mainly enhances metabolite upregulation, whereas *chuT* primarily contributes to downregulation, *chuT* and *shuU* deletions stimulate host immune responses and alter energy metabolism, emphasizing complex host–pathogen interactions in the honeybee gut. Thresholds: *R* > 0.91, *P*‐value < 0.05.

In this research we employed transcriptome and nontargeted metabolite sequencing to generate dual‐omics data, harvesting the honeybee gut tissue transcriptome and metabolome. We identified 12,797 transcripts and 1341 metabolites in total. Using the r package wgcna, we conducted a dual‐omics Pearson/Spearman correlation analysis (*R* > 0.91, *P*‐value < 0.05) to assess the interaction between genes and metabolites, with results depicted in nine‐quadrant plots (Fig. 5). The analysis suggested that *chuT* and *shuU* deletions significantly impact LF82 pathogenicity, with *chuT* alterations causing more pronounced correlation variations and *shuU* deletions being potentially more critical for LF82 virulence. The *shuU* gene appears to play a greater role in positively regulating metabolite upregulation, while *chuT* is more implicated in negative regulation (Fig. 5A–D). In brief, the deletion of pathogenic bacterial genes *chuT* and *shuU* may trigger host innate immune responses and disrupt normal energy metabolism, highlighting the intricate host‐pathogen interactions within the honeybee gut.

## Discussion

In this work we utilized the pathogenic *E. coli* LF82 strain to probe the molecular mechanisms of pathogenicity associated with the *chuT* and *shuU* genes. Specifically, the *chuT* gene in *E. coli* LF82 encodes the heme ABC transport substrate‐binding protein, while *shuU* is responsible for the heme ABC transport permease. The honeybee (*A. mellifera*) serves as an ideal model organism because of advantages, such as simplistic gut microbiota, the feasibility of creating GF models, and the ability to establish monoculture colonization.

In this study we successfully constructed the *chuT* deletion strain LF82∆chuT and the *shuU* deletion strain LF82∆shuU using the CRISPR‐Cas9 system, in which the *chuT* gene carried a 530 bp fragment deletion and the *shuU* gene carried a 450 bp fragment deletion. Utilizing the pEcgRNA vector as a scaffold, we constructed recombinant plasmids harboring gRNAs and donor DNA based on the pEcCas/pEcgRNA system developed by Yang and colleagues [16]. These plasmids were then transformed into bacterial cells to achieve targeted gene editing.

Our colonization experiments with the K12 MG1655, LF82, LF82∆chuT, and LF82∆shuU strains in the gut of sterile honeybees aimed to elucidate the underlying causes of pathogenesis. The results revealed that the nonpathogenic MG1655 group exhibited enhanced survival compared to the pathogenic strains, aligning with previous findings [14]. Surprisingly, our results showed that the median survival time of honeybees in the LF82ΔchuT and LF82ΔshuU cohorts was reduced compared to the LF82 control group. Additionally, no significant survival differences were observed between the LF82∆chuT and LF82∆shuU groups. Statistically significant differences in survival curves were observed exclusively in the LF82ΔshuU group when compared with the LF82 group (*P* < 0.05). These outcomes deviated from our initial theoretical projections. We contemplated several potential explanations for this discrepancy. First, the study was principally aimed at unraveling the pathogenic mechanisms exerted by the LF82 strain on the host bees after pivotal gene deletions. Second, the longevity of the bees under experimental conditions is a multifactorial consequence, influenced by their genetic background, nutritional status, and the profile of gut microbiota. Third, our transcriptomic analysis showed that genes upregulated in both the LF82ΔchuT and LF82ΔshuU groups were predominantly associated with the longevity regulatory pathway. This suggests that the ablation of key genes in the *E. coli* LF82 strain induces changes in bacterial activity, which in turn modulates hosts' expression of genes implicated in longevity regulation. The literature has reported that factors such as mitochondrial function, oxidative stress, and the insulin‐like signaling pathway influence the lifespan of species [17]. While heightened oxidative stress can lead to an overproduction of reactive oxygen species (ROS), inciting inflammation and cellular damage, empirical evidence from model organisms, including nematodes, *Drosophila*, and mice, indicates that a certain degree of mitochondrial dysfunction can paradoxically enhance lifespan, a phenomenon described as “mitohormesis” [18, 19, 20]. We speculate that the LF82ΔshuU strain, following the deletion of *shuU*, may exert a comparatively subdued negative stimulus on the host, resulting in lower intracellular oxidative stress and ROS levels, insufficient to trigger the “mitohormesis.” Conversely, the LF82 and LF82ΔchuT strains may activate this effect to some extent, potentially accounting for the reduced lifespan of bees in the LF82ΔshuU group relative to the LF82 group. To thoroughly elucidate the intricate mechanisms underpinning this phenomenon, future research endeavors are imperative.

Our study assessed the impact of *E. coli* strains on the intestinal permeability of the host, *A. mellifera*, and observed statistically significant differences (*P* = 0.041). However, no significant difference in permeability was detected between the LF82∆chuT and LF82∆shuU groups (*P* > 0.05). Nevertheless, the LF82∆shuU group exhibited a lower smurf positive rate, suggesting that the deletion of the *shuU* gene may result in reduced intestinal damage in honeybees compared to the deletion of *chuT*, potentially indicating a greater role for *shuU* in pathogenicity. The absence of significant permeability differences between the groups could be attributed to variability in honeybee cohorts. This aligns with previous findings that *E. coli* LF82 infection can induce inflammatory changes in the intestines of honeybees and zebrafish, corroborating the consistency of our results with the existing literature [14, 21].

In this investigation we conducted a comprehensive analysis of the transcriptome and untargeted metabolome of honeybee intestinal tissues colonized by various *E. coli* strains. This approach allowed us to observe the effects of key gene deletions on the pathogenicity of bacteria and the corresponding host response, as well as to elucidate the molecular mechanisms driving specific gene expression and metabolite alterations associated with disease states.

Hosts have developed a myriad of defense mechanisms, such as “nutritional immunity,” to thwart pathogen infections. Iron, a critical nutrient for bacterial survival, is implicated in numerous physiological processes, including DNA synthesis, damage repair, and oxidative stress response. While heme‐derived iron is vital for bacterial virulence, excessive iron uptake can be detrimental to bacteria [8], necessitating a delicate balance in iron homeostasis for bacterial survival and proliferation [7].

Previous research by Sassone‐Corsi *et al*. demonstrated that siderophores could inhibit *Salmonella* colonization [22]. Similarly, Gerner *et al*.'s work indicated that iron siderophores in mice led to reduced intestinal colonization by AIEC, mitigating colitis by diminishing AIEC interactions with the intestinal mucosa [23]. These findings suggest that siderophores could be viable therapeutic targets to curb the progression of colitis by limiting AIEC colonization and interaction with the intestinal lining. GO enrichment analysis of the transcriptome revealed that the LF82∆chuT and LF82∆shuU groups exhibited upregulation of genes associated with iron ion binding, heme binding, redox processes, and tetrapyrrole binding compared to the nonpathogenic MG1655 group. Conversely, these groups showed downregulation of genes related to iron and heme binding when compared to the LF82 group. These observations imply that the deletion of *chuT* and *shuU* in *E. coli* LF82 diminishes iron uptake, indirectly influencing host iron and heme metabolism and indicating a dynamic interplay between bacterial iron acquisition and host response. Iron is an essential micronutrient for nearly all organisms, and free iron ions are normally low in human. Pathogenic gut microbes have developed sophisticated iron acquisition strategies, while hosts have evolved countermeasures to restrict bacterial access to iron, thereby defending against pathogenic invasions [24].

Oxidative stress plays a pivotal role in the pathogenesis of IBD, with a profound connection to its development and clinical symptoms [25]. Our transcriptomic data corroborate previous findings, presenting enrichment in GO terms related to redox processes. In IBD patients, oxidative stress is not confined to the inflamed mucosa, but can extend deeper into the intestinal wall and even invade the body circulation [26, 27]. The persistent impact of ROS on the inflamed mucosa can impair normal gastrointestinal function [28, 29]. When compared to the MG1655 group, the LF82 strain and its deletion mutants, LF82∆chuT and LF82∆shuU, exhibited upregulated gene enrichment in oxidation–reduction processes, oxidoreductase activity, and carbohydrate metabolism, suggesting a heightened oxidative stress response in these pathogenic and mutant strains.

Recent advances have highlighted the autophagy‐lysosome pathway as a crucial mediator of oxidative stress [30]. Our data, supported by KEGG enrichment analysis, indicate that the *E. coli* LF82 strain and its gene deletion mutants, LF82∆chuT and LF82∆shuU, upregulated genes associated with the lysosome pathway compared to the MG1655 group. This suggests that *E. coli* LF82 and its mutants activate host lysosomal reactions and cellular redox activities, potentially as a response to oxidative stress. Furthermore, the *E. coli* LF82 strain and its mutants appear to stimulate the host's Toll and Imd signaling pathways, which are known to trigger defense responses, including the secretion of antimicrobial peptides [31].

The structural integrity of the intestinal mucosal barrier is fundamental for nutrient metabolism and immune response, and its impairment is implicated in the pathogenesis of IBD [29]. Elevated intestinal permeability, sometimes preceding clinical symptoms, has been observed in both animal models and patients with IBD. In patients with colitis, there is an increase in the secretion and chemical modification of mucopolysaccharides and glycosaminoglycans (GAGs), enhancing the intestinal defense barrier. Lee *et al*.'s research suggests that intestinal flora may exacerbate colitis by degrading GAGs, and that antibiotics could mitigate colitis by inhibiting the growth of GAG‐degrading bacteria [32, 33]. In line with these findings, KEGG enrichment analysis revealed that the *E. coli* LF82 strain and its mutants upregulated genes involved in the glycosaminoglycan degradation pathway compared to the MG1655 group. This indicates that they may contribute to the increased degradation of host GAGs, potentially leading to inflammation and damage to the intestinal mucosal barrier. Our study also demonstrates that *E. coli* LF82 and its mutants stimulate the innate immune system of the host, eliciting defense responses as well as inflammatory and redox reactions. Notably, the upregulated genes in the LF82∆chuT and LF82∆shuU groups were enriched in pathways related to tryptophan metabolism and fatty acid metabolism, whereas the LF82 group did not show enrichment in these pathways. This differential enrichment may reflect the nuanced effects of gene deletions on the host's metabolic response to bacterial colonization.

Complementing intestinal transcriptomic analysis, we employed nontargeted metabolomics to discern variations in intestinal metabolites across the experimental groups. The KEGG pathway enrichment analysis of differential metabolites revealed that the LF82 group's metabolites were predominantly associated with tryptophan metabolism, galactose metabolism, and propanoate metabolism when compared to the MG1655 group. In contrast, the metabolites of the LF82∆chuT and LF82∆shuU groups were notably linked to tyrosine metabolism and galactose metabolism, among others. These findings indicate that *E. coli* LF82 and its variants can induce alterations in the host's intestinal metabolism of tryptophan and tyrosine.

Tryptophan, an essential aromatic amino acid, is metabolized in the intestine through three primary pathways: serotonin (5‐hydroxytryptophan), kynurenine (Kyn), and indole derivatives, all of which are directly or indirectly modulated by the gut microbiota. Disruptions in tryptophan metabolism are increasingly recognized for their potential role in the pathogenesis of IBD [34]. Li *et al*.'s research demonstrated that the tryptophan metabolite indole‐3‐propionic acid enhances intestinal barrier function by augmenting tight junctions, mucin production, and cuprocyte secretion products, while synergistically modulating multiple inflammatory cytokines and the PI3K/AKT/mTOR signaling pathway *in vitro* [35]. Studies in murine and porcine models have shown that dietary supplementation with tryptophan ameliorates symptoms of dextran sodium sulfate‐induced colitis, improves intestinal permeability, and attenuates local inflammatory responses [36]. Furthermore, Nikolaus *et al*. reported a negative correlation between serum tryptophan levels and disease activity, underscoring the significance of tryptophan metabolism in the clinical manifestations of IBD [37]. Collectively, these insights suggest that the observed metabolic shifts in tryptophan and tyrosine pathways may have profound implications for the host's intestinal health and immune response in the context of pathogenic *E. coli* colonization.

Beyond tryptophan metabolism, glutathione (GSH) plays a vital role in counteracting oxidative stress. Research has shown that some patients with IBD exhibit reduced activity of key enzymes involved in GSH synthesis and a deficiency in cysteine, the substrate for GSH synthesis, leading to mucosal GSH depletion. This impairment in mucosal antioxidant capacity may exacerbate oxidative damage. Hence, GSH deficiency may be one of the targets for therapeutic intervention in IBD [38].

Previous research indicates that the gut microbiome in IBD patients is more enriched in genes for oxidative stress response and nutrient transport, yet less so for amino acid synthesis and carbohydrate metabolism [39, 40]. In the current study, the LF82∆chuT and LF82∆shuU groups showed differential metabolite enrichment in redox metabolic pathways. Based on the above results, we deduce that the loss function of the *chuT* and *shuU* genes may alleviate intestinal oxidative stress through modulation of the glutathione metabolic pathway.

In the present study we utilized transcriptome sequencing and nontargeted metabolite profiling to generate bi‐omics data, encompassing both the honeybee intestinal tissue transcriptome and the metabolome of the intestinal contents. We identified a total of 12,797 transcripts and 1341 metabolites. Compared to the MG1655 strain, the LF82 strain not only disrupted host energy metabolism, but also elicited a more robust immune response, leading to abnormal purine and uric acid metabolism in the host honeybee gut. This is in line with previous findings of decreased purine levels and increased uric acid levels in murine models, as well as with the disturbed purine metabolism observed in IBD patients.

We acknowledge the limitations inherent in employing the honeybee model for investigating mechanisms of human CD, particularly considering the intricate interaction between microbial virulence and host pathogens. Although honeybees display significant physiological and evolutionary differences from mammals, they provide the benefits of a simple gut microbiota and the ability to rapidly generate sterile models [15]. These features render them an effective model for probing fundamental host–pathogen dynamics. This work focuses on the interaction mechanisms between *E. coli* LF82, its gene deletion mutants, and the host (honeybee), with a particular emphasis on elucidating the potential molecular mechanisms of pathogenesis involving the *chuT* and *shuU* genes through iron uptake pathways and genes. These results contribute to the groundwork for additional detailed studies on the pathogenic mechanisms in mammalian models, supplying valuable omics data to enhance our understanding of CD pathogenesis.

*Escherichia coli* LF82, coupled with its mutant strains LF82∆chuT and LF82∆shuU, not only elicit host inflammatory and immune responses, but also compromise the integrity of the intestinal barrier through the enhanced degradation of glycosaminoglycans, and perturb host energy metabolism. Critically, the deletion of *chuT* or *shuU* genes in *E. coli* LF82 induces changes in host iron homeostasis, leading to significant alterations in both iron and heme metabolic pathways. Furthermore, mutants devoid of *chuT* or *shuU* spur modifications in host tryptophan metabolism, which plays a role in alleviating intestinal inflammation. Intriguingly, our integrative dual‐omics analysis indicates that the *shuU* gene holds a more pivotal role in the iron acquisition of pathogenic bacteria compared to the *chuT* gene. These findings suggest that restricting iron transport in pathogenic bacteria could be a strategy to reduce their virulence. We propose that the bacterial loss of *chuT* or *shuU* genes may alleviate intestinal oxidative stress and intestinal inflammation, partially through the modulation of tryptophan metabolism. Additional research in the future is necessary to conduct a more comprehensive and in‐depth analysis of the intricate mechanisms underlying this issue.

## Conflict of interest

The authors declare no conflicts of interest.

### Peer review

The peer review history for this article is available at https://www.webofscience.com/api/gateway/wos/peer‐review/10.1002/2211‐5463.13776.

## Author contributions

YY conceived and supervised the study. DF, HZ, ZL, JY, and YL conducted the experiments. DF and HZ conducted bioinformatic analysis. YZ and YY performed project administration. DF and HZ wrote the article. YY analyzed and reviewed the article.

## Supporting information


**Fig. S1.** The original experimental data of plasmid curing in Fig. 1E.

**Table S1.** Primers used.

## Data Availability

The datasets presented in this study can be found in the online repositories. The names of the repository/repositories and accession number(s) can be found in the article/[Link], [Link]. The data of transcriptome and metabolome generated in this study have been deposited with the National Genomics Data Center (China Nation Center for Bioinformation) with accession number: PRJCA019138.
