# Supplementary material for: Categorization of the effects of E. coli LF82 and mutants lacking the chuT and shuU genes on survival, the transcriptome, and metabolome in germ‐free honeybee
Source: FEBS Open Bio. 2024 Feb 25;14(5):756–70. doi: 10.1002/2211-5463.13776 (PMC11073505; doi:10.1002/2211-5463.13776)
Supplement: Supplementary file 1 — Fig. S1. The original experimental data of plasmid curing in Fig. 1E. [file FEB4-14-756-s001.docx]

**（A）**

**（B）**

Figure S1. The original experimental data for plasmid curing in Figure1E.

(A) pEcgT-LF82-∆chuT and pEcCas were removed in id 1 to 8, 11, 13, 14, 18, 20, 22 to 30 after *chuT* gene knockout. (B) pEcgT-LF82-∆chuT and pEcCas were removed in id 1, 3, 4, 5, 8, 9, 10, 12,13,15, 16, 17, 19 to 30 after *shuU* gene knockout.
